# Treatment-duration is related to changes in peripheral lymphocyte counts during definitive radiotherapy for unresectable stage III NSCLC

**DOI:** 10.1186/s13014-019-1287-z

**Published:** 2019-05-27

**Authors:** Qianqian Zhao, Gang Chen, Luxi Ye, Shiming Shi, Shisuo Du, Zhaochong Zeng, Jian He

**Affiliations:** Department of Radiation Oncology, Zhongshan Hospital, Fudan University, 180 Fenglin Road, Shanghai, 200032 China

**Keywords:** Non-small cell lung cancer, Total lymphocyte counts, Fractionation regimes, Overall treatment time

## Abstract

**Background:**

To investigate the potential impact of fractionation regimes and overall treatment time (OTT) on lymphopenia during definitive radiotherapy (RT) and its associations with patient outcomes in non-small cell lung cancer (NSCLC).

**Methods:**

Subjects consisted of 115 patients who had received definitive chemoradiation therapy (CRT) with different doses and fractions for unresectable stage III NSCLC. Clinical and laboratory records were reviewed to assess the changes in total lymphocyte counts (TLCs) during definitive RT. The associations of the TLCs with the clinical and treatment features, and outcomes were analyzed.

**Results:**

The median reduction of TLCs in the entire cohort was 1300 cells/μL (interquartile range [IQR], 950–1510 cells/μL). Of all patients, 63 (54.8%) experienced severe lymphopenia (SL) (TLC nadir < 500 cells/μL), which occurred at a median of the 5th week following RT initiation, not at the completion of RT or upon treatment with maximal doses. SL risk was increased over the first 5 weeks (odds ratio [OR] = 3.455, *P* = 0.007), after which, no increased risk was observed (OR = 0.562, *P* = 0.216). The median TLCs remained low and failed to recover to the initial normal values of their pre-RT level after 2 months of RT completion. Patients without SL exhibited significantly improved progression-free survival (hazard ratio [HR] = 0.544, *P* = 0.010) and overall survival (HR = 0.463, *P* = 0.011) after controlling for confounding variables in multivariate analyses. The incidence of SL was significantly lower (71.1% reduction in risk (OR = 0.289, *P* = 0.007)) in patients who received hypofractionated RT with an OTT within 4 weeks, compared to those who had an OTT of more than 4 weeks (32.1% vs 62.1%, *P* = 0.006). Multivariate analyses revealed that OTT within 4 weeks (OR = 0.322, *P* = 0.032) was significantly associated with a decreased risk of developing SL after controlling for confounding factors.

**Conclusions:**

Hypofractionated RT was significantly associated with a decreased risk of SL and improved survival during definitive radiotherapy for unresectable stage III NSCLC.

**Electronic supplementary material:**

The online version of this article (10.1186/s13014-019-1287-z) contains supplementary material, which is available to authorized users.

## Background

Definitive radiotherapy (RT) with platinum-based doublet chemotherapy has been widely recognized over the past few decades as the standard of care for unresectable stage III non-small cell lung cancer (NSCLC). However, the survival outcomes with this approach remain disappointing, with a 5-year overall survival (OS) rate of approximately 15% using conventionally-fractionated radiotherapy (CFRT) with doses of 60–66 Gy in 30–33 fractions [1, 2]. As promising results were observed in the PACIFIC trial, consolidation treatments with immune checkpoint inhibitors (ICIs) are now recommended for patients with stage III, unresectable NSCLC after receiving definitive CRT [3]. However, on the basis of CFRT, increasing the number of fractions and radiation duration to increase the overall radiation dose did not improve outcomes for patients [4]. With higher biological effective dose (BED) over a shorter period, hypofractionated radiotherapy (HFRT) could overcome the accelerated repopulation of tumor cells during treatment and theoretically obtain better efficacies [5, 6]. However, the clinical application of HFRT has been limited due to the side effects of irradiation, particularly grade III or IV toxicities [6]. In recent years, advances in RT techniques have facilitated the safe and effective delivery of high doses of radiation to tumors while preserved the surrounding disease. Several studies reported that HFRT could achieve good local control with well-tolerated toxicity for locally-advanced NSCLC when modern RT techniques were utilized [7–9]. HFRT is experiencing increasing importance in the treatment of locally-advanced NSCLC.

Numerous studies demonstrated that the host immune system and peripheral lymphocyte populations play crucial roles in oncologic outcomes, and the reduction in total peripheral lymphocyte counts (TLCs) results in impairing antitumor immunity [10, 11]. Lymphopenia is a common consequence of RT in cancer patients, regardless of whether other lymphotoxic agents, such as corticosteroids or cytotoxic chemotherapeutics are administered[12–15]. The preclinical model suggested that decreasing the target radiation volume and fraction size significantly reduced the circulating blood exposure radiation dose [16]. It is therefore possible that protracted radiation courses with higher fractions will increase exposures to radiation and increase lymphocyte destruction. Such assumptions have led some researchers to conclude that HFRT with shorter treatment periods would reduce radiation exposure dose to circulating blood and spare peripheral lymphocytes, compared to CFRT with treatment period extending.

Despite theoretical superiority, the impact of different fractionation regimes and overall treatment times (OTT) on radiation-induced lymphopenia (RIL) in unresectable stage III NSCLC was largely unexplored until recently. In our institution, apart from CFRT, other fractionation regimes with increased doses per fraction and shorter OTT have been increasingly adopted since 2011 for locally advanced NSCLC. Therefore, we sought to evaluate the potential impact of fraction regime and OTT on TLCs by retrospectively analyzing stage III, locally advanced, unresectable NSCLC patients receiving definitive chemoradiation therapy (CRT) at our institution.

## Methods

### Patient population

After approval by the institutional review board, we retrospectively analyzed the medical records of consecutive patients with histologically-verified NSCLC receiving definitive CRT (equivalent dose in 2 Gy fraction [EQD2] ≥ 60 Gy, whereas the α/β ratio for lung cancer is 10 Gy [16]) at Zhongshan Hospital, Fudan University from January 2011 to December 2017. All patients were (re)staged according to the 8th edition of the AJCC TNM classification. The inclusion criteria were as follows: (1) unresectable Stage III NSCLC;(2) age 18 years or above and an Eastern Cooperative Oncology Group (ECOG) performance status ≤2; (3) no history of a concomitant malignancy; (4) no prior RT or immunotherapy for any disease; (5) finished the scheduled treatment including four or more cycles of platinum-based doublet chemotherapy; (6) complete and retrievable medical records, including at least 3 documented weekly TLCs values during RT course were available for review. Patients were excluded if they had received any other anticancer treatments besides CRT before disease progression, had used any systemic corticosteroids during RT except for pretreatment before chemotherapy, had any ongoing infection, rheumatoid disease, and had immune system disease. All patients underwent a comprehensive assessment within 3 weeks prior to the treatment, including routine blood testing, pulmonary function tests, chest/abdominal CT with contrast, brain magnetic resonance imaging, abdominal ultrasonography, bone scan, and/or positron emission tomography/computed tomography (PET/CT). Informed consent was obtained from all patients.

### Therapeutic interventions

Treatment planning, including radiation targets, normal tissue dose constraints, and delivery techniques for locally advanced NSCLC in our institution have been previously described [8]. The fractionation regimes primarily depended on the treating physicians’ preference, based on the clinical tumor size and location. Typically, 2.0 to 3.0 Gy per fraction for 20–30 fractions, and a total dose of 60–70 Gy were adopted in our institution. The fractionation regimes information used in our study population were shown in Table 1. The median calculated BED was 76.8 Gy (interquartile range [IQR] 72.0–78.0 Gy), whereas the α/β ratio for lung cancer is 10 Gy [17], and the median EQD2 was 64.0 Gy (IQR, 60.0–65.0Gy).

**Table 1:** Details of fractionation regimes used in our study population (*n* = 115)

Fractionation regime	No. of cases (%)
6000 cGy/20 fractions	30 (26.1%)
5940 cGy/22 fractions	2 (1.7%)
6300 cGy/23 fractions	1 (0.9%)
6000 cGy/24 fractions	1 (0.9%)
6240 cGy/24 fractions	1 (0.9%)
6000 cGy/25 fractions	11 (9.6%)
6100 cGy/25 fractions	1 (0.9%)
6250 cGy/25 fractions	9 (7.8%)
6400 cGy/25 fractions	1 (0.9%)
6500 cGy/25 fractions	1 (0.9%)
7000 cGy/25 fractions	1 (0.9%)
6240 cGy/26 fractions	1 (0.9%)
6210 cGy/27 fractions	4 (3.5%)
6160 cGy/28 fractions	5 (4.3%)
6000 cGy/30 fractions	30 (26.1%)
6400 cGy/32 fractions	8 (7.0%)
6600 cGy/33 fractions	6 (5.2%)
7000 cGy/35 fractions	2 (1.7%)

Note: The prescribed doses were given to gross tumor volume

### Data acquisition

Clinical and laboratory parameters were extracted from the electronic medical record for all patients. Patient-specific variables included age, sex, ECOG performance status, smoking history, and baseline laboratory values. Tumor-specific variables included histological type and TNM classification. Treatment-specific variables included the RT technique, dose per fraction, fractions, treatment interruption, receipt of chemotherapy, chemotherapy type, and chemotherapy timing. Dosimetric parameters included gross target volume (GTV), planning target volume (PTV), mean dose to lung (MLD) and heart (MHD), and percentage volume of lung and heart receiving 5–60 Gy (V5–60 Gy) in 5 Gy increments, respectively.

TLCs were collected and investigated at the following intervals: within 2 weeks prior to induction chemotherapy or RT (baseline TLCs), within 2 weeks prior to the start of RT (Pre-RT TLCs), weekly during radiation treatment, and 1 and 2 months after RT completion. If complete blood cell counts were not available at the exact time point, i.e. no TLCs data were recorded for that time point. TLCs nadir was defined as the minimum value of TLCs during the course of RT. Severe lymphopenia (SL) was defined as TLCs nadir below 500 cells/μL during the course of RT, consistent with grade 3 toxicity according to the Common Terminology Criteria for Adverse Events version 5.0.

Clinical follow-up was generally conducted every 3 months for the first year, every 6 months for the next 2 years, and yearly thereafter. Follow-up examinations included physical examinations, blood tests, chest CT scans, and/or PET/CT. Further examinations were performed when needed for clinical purposes. Progression-free survival (PFS) was calculated from the date of pathologic diagnosis to the date of disease progression, death or last follow-up. OS was calculated from the date of pathologic diagnosis to the date of death from any cause or last follow-up. Patients were censored at the date of the last available follow-up if alive and the data were updated in October 2018.

### Statistical analysis

Patients, tumor, and treatment characteristics for the entire group and patient subgroups were summarized using descriptive statistics. To visualize trends in TLCs over the course of RT, TLCs were plotted versus RT time (weeks). Chi-squared and non-parametric tests were used to compare the differences in proportions or medians between groups. Survival rates were calculated using the Kaplan-Meier method, and comparisons were made using the log-rank test. Prognostic variables with *p*-values ≤0.2 on univariate analyses were entered as covariates in the multivariable Cox proportional hazards model using backward stepwise selection. Spearman correlations were used to examine correlations between dosimetric parameters and TLC nadir. Receiver operating characteristic (ROC) curve analyses were used to determine the optimal cut-off points for continuous variables identified as influencing SL. Univariate logistic regression analyses were conducted to identify variables potentially associated with an increased risk of SL. Multivariate stepwise logistic regression (using variables with univariate significance of *p* ≤ 0.2) were performed to assess the independent effect of such factors on the development of SL. In addition, age, sex, and ECOG performance status were retained in the initial multivariable model owing to their perceived clinical significances. All statistical analyses were conducted using IBM SPSS software version 23.0 (SPSS Inc., Chicago, IL, USA). Statistical tests were two-sided, and a *p*-value < 0.05 was considered statistically significant.

## Results

### Patient characteristics

A total of 115 patients met the inclusion criteria and were enrolled in the study. Baseline demographic, tumor, and treatment characteristics of all eligible patients are listed in Table 2. The median number of chemotherapy cycles was six (interquartile range [IQR], 4–6 cycles). Concurrent CRT regimens were given to 31 patients (27.0%), and sequential CRT were given to 84 patients (73.0%). Additional details are provided in (Additional file 1: Table S1). Approximate 80% patients had received induction chemotherapy and they all had the baseline TLCs data. All patients had the Pre-RT TLCs. During the RT duration, all patients had at least 3 documented weekly TLCs values available for review. In the follow-up duration, approximate 70% patients had the TLCs data at 1 month and 63% patients had the TLCs data at 2 months. No significant differences in the median TLC nadir existed between patients who received sequential or concurrent CRT (500 vs 560 cells/μL; *P* = 0.725). As noted in Table 2, the SL subset was significantly different from the non-SL subset in lower baseline TLCs, larger GTV, and higher radiation doses to the lungs and heart.

**Table 2:** Baseline patient, tumor, and treatment characteristics for all patients (*n* = 115) and broken down by patients did or did not experience SL

Characteristic	Total (*N* = 115)	SL (*N* = 63)	No SL (*N* = 52)	*P* value
Age at diagnosis, years, Median (IQR)	63.00 (59.00–67.00)	63.00 (60.00–67.00)	62.50 (55.50–67.00)	0.362
Sex				
Male	107 (92.2%)	59 (51.3%)	48 (41.7%)	
Female	8 (7.0%)	4 (3.5%)	4 (3.5%)	0.778
ECOG performance status				
0–1	84 (73.0%)	44 (38.3%)	40 (34.8%)	
2	31 (27.0%)	19 (16.5%)	12 (10.4%)	0.394
Smoking pack-years				
≥ 10	80	45	35	
0–10	4	3	1	
0	24	12	12	
Unkown	7	3	4	0.717
Histology				
Squamous carcinoma	78 (67.8%)	49 (42.2%)	35 (30.4%)	
Adenocarcinoma	34 (29.6%)	19 (16.5%)	15 (13.0%)	
Others	3 (2.6%)	1 (0.9%)	2 (1.7%)	0.683
cT stage (AJCC 8th ed)				
T1	14 (12.2%)	8 (7.0%)	6 (5.2%)	
T2	31 (26.7%)	20 (17.2%)	11 (9.57%)	
T3	27 (23.5%)	12 (10.4%)	15 (13.0%)	
T4	43 (37.4%)	23 (20.0%)	20 (17.4%)	0.492
cN stage				
N1	6 (5.2%)	2 (1.7%)	4 (3.5%)	
N2	73 (63.5%)	39 (33.9%)	34 (29.6%)	
N3	36 (31.3%)	22 (19.1%)	14 (12.2%)	0.417
Stage				
IIIA	33 (28.7%)	18 (15.7%)	15 (13.0%)	
IIIB	61 (53.0%)	33 (28.7%)	28 (24.3%)	
IIIC	21 (18.3%)	12 (10.4%)	9 (7.8%)	0.971
TLCs, cells/μL (median, IQR)				
Baseline	1600 (1400–2000)	1600 (1300–1900)	1800 (1400–2200)	0.037
Pre-RT	1800 (1400–2100)	1600 (1280–1900)	1965 (1700–2252)	< 0.001
TLC nadir during the course of RT	500 (380–700)	390 (300–470)	700 (600–830)	< 0.001
1 month after RT	915 (700–1100)	800 (598–1100)	995 (882–1232)	0.004
2 month after RT	900 (700–1100)	740 (600–982)	1000 (810–1195)	< 0.001
Tumor volume, cm^3^ (median, IQR)				
GTV	56.85 (32.47–129.35)	87.69 (45.58–144.92)	43.40 (21.84–95.52)	0.006
PTV	146.03 (74.20–247.39)	160.95 (80.69–258.92)	124.82 (71.13–224.05)	0.192
EQD2 (Gy or Gy[RBE]) (median, IQR)	64.00 (60.00–65.00)	62.87 (60.00–65.10)	65.00 (60.00–65.00)	0.755
BED (Gy)	76.80 (72.00–78.00)	75.44 (72.00–78.13)	78 (72–78)	0.768
Fractions, number (median, IQR)	25 (20–30)	25 (25–30)	25 (20–30)	0.141
OTT, days (median, IQR)	39 (31–43)	39 (33–43)	35 (29–43)	0.281
Key mean normal tissue doses, cGy (median, IQR)				
Mean lung dose	1275 (1078–1454)	1354.90 (1146.00–1480.00)	1165 (990–1386)	0.007
Mean heart dose	601 (420–1261.50)	886 (439.00–1502.25)	518 (384–936.90)	0.041
Radiation technique				
3D conformal	28 (24.3%)	16 (13.9%)	12 (10.4%)	
Intensity modulated	37 (32.2%)	19 (16.5%)	18 (15.7%)	
Helical Tomotherapy	50 (43.5%)	28 (24.3%)	22 (19.1%)	0.874
Concurrent chemoradiation (sequential)				
Yes	31 (27.0%)	15 (12.9%)	16 (13.9%)	
No	84 (73.0%)	48 (41.7%)	36 (31.3%)	0.403
Induction chemotherapy				
Yes	91 (79.1%)	54 (47.0%)	37 (32.2%)	
No	24 (20.9%)	9 (7.8%)	15 (13.0%)	0.056
Adjuvant chemotherapy				
Yes	61 (53.0%)	31 (27.0%)	30 (26.1%)	
No	54 (47.0%)	32 (27.8%)	22 (19.1%)	0.364

*Abbreviations*: *SL* severe lymphopenia, *IQR* interquartile range, *ECOG* Eastern Cooperative Oncology Group, *AJCC* American Joint Committee on Cancer, *TLCs* total peripheral lymphocyte counts, *GTV* gross tumor volume, *PTV* planning target volume, EQD2 equivalents (relative biological equivalents), *BED* biological effective dose, *OTT* overall treatment time

### TLCs trend over the time

**Fig. 1:**
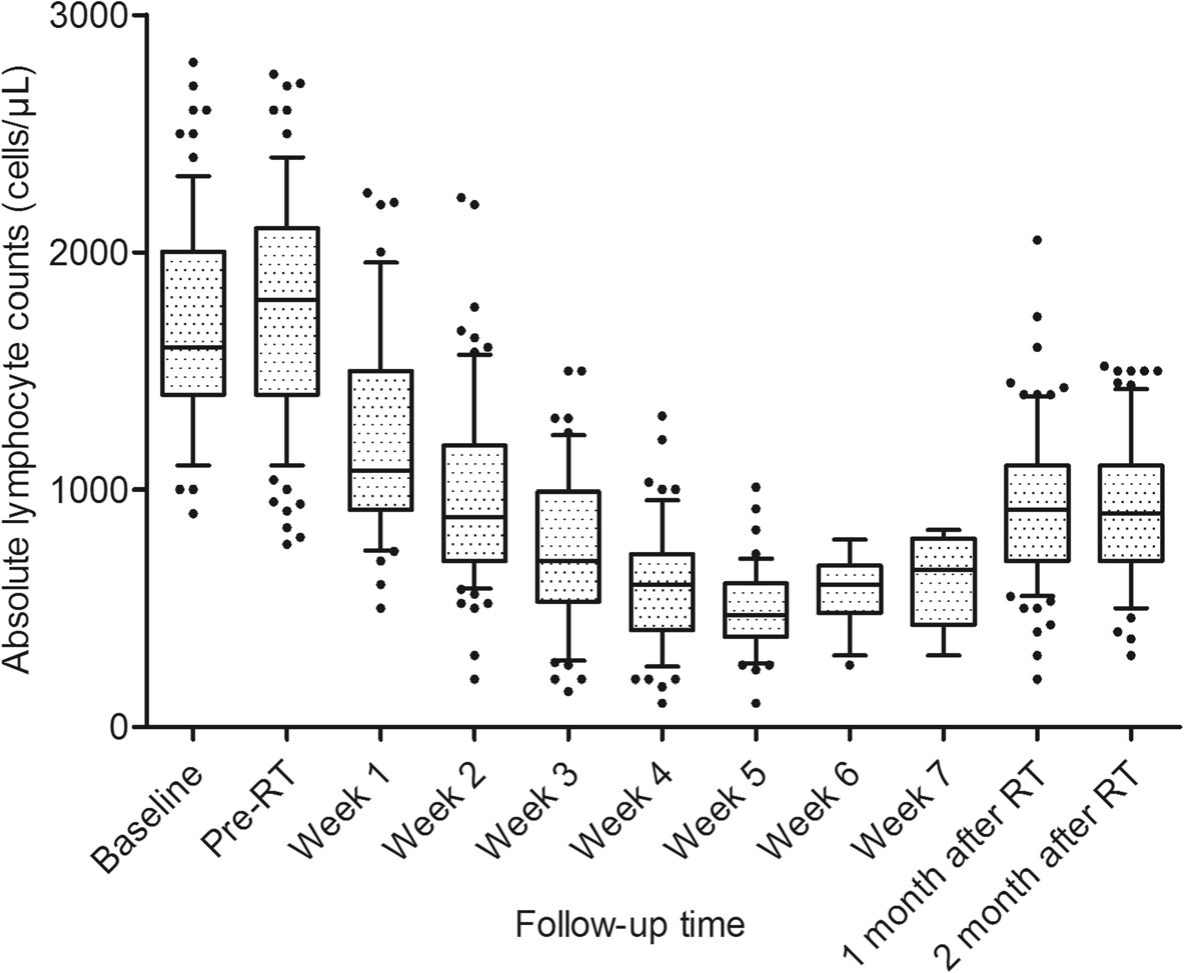
Total lymphocyte counts (TLCs) over time. Baseline corresponds to within 2 weeks prior to induction chemotherapy or radiotherapy (RT). Pre-RT corresponds to within 2 weeks prior to the start of RT. Week 1–7 corresponds to weekly during-radiation treatments. One and 2 months after RT correspond to 1 month and 2 months following RT completion

Figure 1 summarizes the changes in TLCs over time. The median pre-RT TLC was 1800 cells/μL (IQR, 1400–2100 cells/μL), which was within the normal range and was not different from the baseline level of 1600 cells/μL (IQR, 1400–2000 cells/μL) (*P* = 0.122). No differences in TLC changes were observed between chemotherapy subgroups, whether stratified by the chemotherapy regimen or cycles (all *P* > 0.05). As shown in Fig. 1, TLCs decreased from the beginning of RT, and declined steeply each week during the first 5 weeks before stabilizing. The median TLCs gradually declined to 1080, 885, 700, 600, 470, 600, and 660 cells/μL from weeks 1 to 7, respectively. The median TLC nadir in the entire cohort was 500 cells/μL (IQR, 380–700 cells/μL) and median reduction of TLCs was 1300 cells/μL (IQR, 950–1510 cells/μL). Of all patients, 63 (54.8%) experienced SL, which occurred at a median of the 5th week (IQR, 4–5 weeks) following RT initiation, not at the completion of RT or upon treatment with maximal doses. The SL risk increased over the first 5 weeks (odds ratio [OR] = 3.455, 95% CI, 1.399 to 8.528; *P* = 0.007), after which, risk no longer continued to increase (OR = 0.562, *P* = 0.216). After 2 months following RT, median TLCs remained low and failed to recover to their pre-RT levels. Patients who developed SL were more likely to continue to have persistent lymphopenia one to 2 months after RT (median: 800 vs 995 cells/μL, *P* = 0.004; median: 740 vs 1000cells/μL, *P* <  0.001; respectively) (Table 2).

To better understand the impact of OTT on TLCs in patients, we divided patients into two groups based on changes in TLCs during RT: the short-term radiotherapy (STRT) group had an OTT of 4 weeks or less (≤ 4 weeks), while the long-term radiotherapy (LTRT) group had an OTT of more than 4 weeks (> 4 weeks). Overall, 28 patients (24.3%) received STRT and 87 patients (75.7%) received LTRT. All patients in the STRT group received 60 Gy/20 fractions over a median of 26 days (IQR, 25–28 days), while those in the LTRT group received a median of 60 Gy (IQR, 60–62.50 Gy)/30 fractions (IQR, 25–30 fractions) over 41 days (36–44 days). The incidence of SL was significantly lower in patients who received STRT than in those receiving LTRT (32.1% vs 62.1%, *P* = 0.006), with a 71.1% reduction in risk (OR = 0.289, 95% CI, 0.117–0.715; *P* = 0.007).

### Survival

Eight patients were excluded from survival analyses due to incomplete follow-up data. Nearly half of the patients (*n* = 49) died during the follow-up period. The median follow-up period from diagnosis was 18 months (IQR, 14 to 26 months) and 20 months (IQR, 14 to 28.5 months) for patients alive at the last follow-up. The median PFS for the entire cohort (*n* = 107) was 11 months (95% CI, 10–12 months) and the median OS was 25 months (95% CI, 20–30 months).

**Fig. 2:**
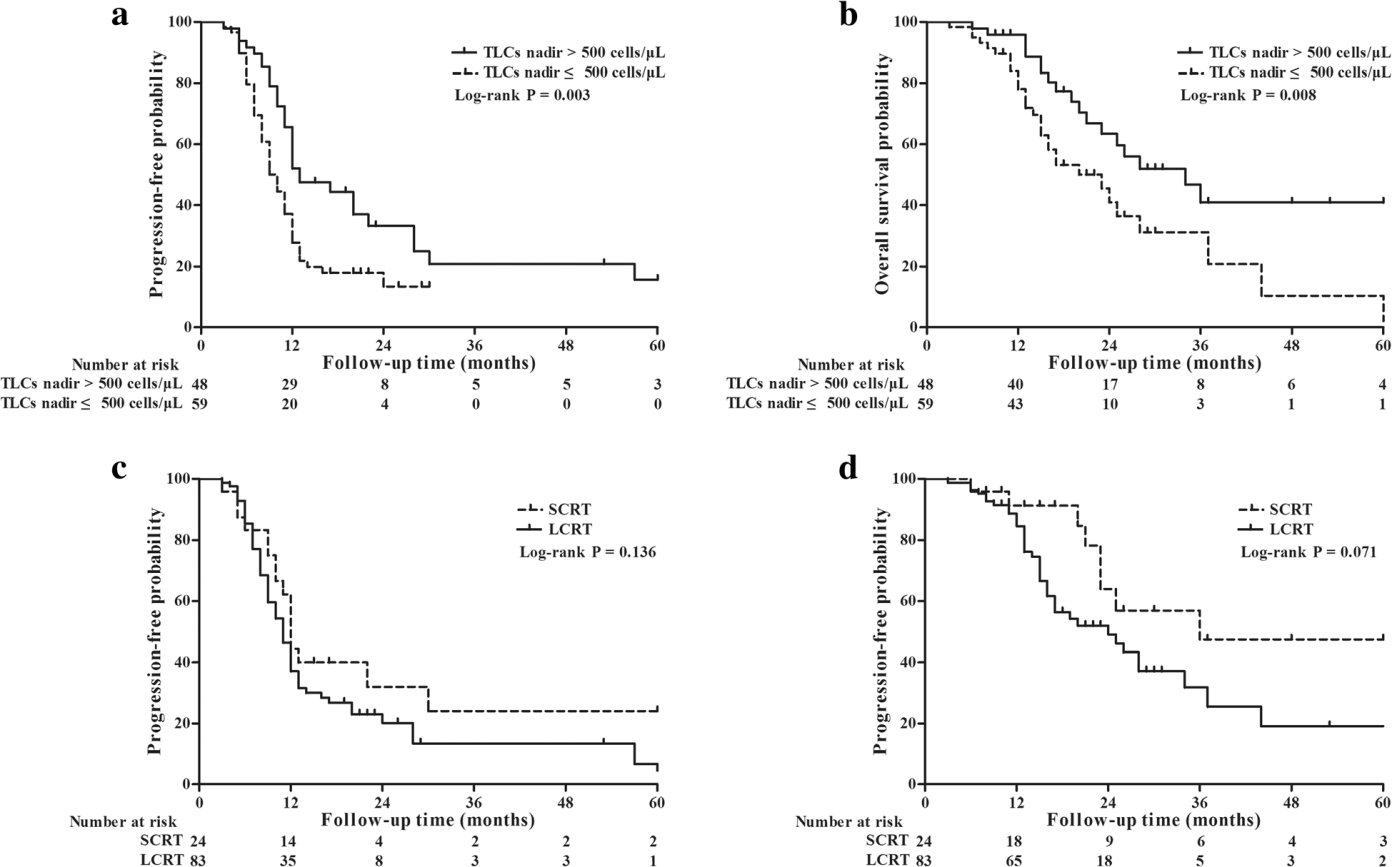
Cumulative Kaplan-Meier plot of survival from the initial pathological diagnosis. Using a TLC nadir of ≤500 as a cutoff, patients with severe lymphopenia during RT had significantly worse progression-free survival (PFS) and overall survival (OS), compared to those who did not (*P* = 0.003, *P* = 0.008, respectively) (**a** and **b**). Patients treated with short-course radiotherapy (overall treatment time was within 4 weeks) had longer median PFS and OS trends than patients with long-course radiotherapy (overall treatment time was more than 4 weeks) (*P* = 0.136, *P* = 0.071, respectively) (**c** and **d**)

Whether the TLC nadir was analyzed as a continuous variable or patients were divided into SL and non-SL groups, they were all associated with PFS (hazard ratio [HR] = 0.999, 95% CI, 0.998–1.000; *P* = 0.012 and HR = 0.524, 95% CI, 0.331–0.831; *P* = 0.006, respectively) and OS (HR = 0.999, 95% CI, 0.997–1.000; *P* = 0.013 and HR = 0.460, 95% CI, 0.255–0.831; *P* = 0.010, respectively). The median PFS was 10 months (95% CI, 8–12 months) in patients who experienced SL and 13 months (95% CI, 9–17 months) in those who did not (*P* = 0.003), while the OS was 23 months (95% CI, 15–31 months) and 34 months (95% CI, 21–47 months), respectively (*P* = 0.008) (Fig. 2a and b). The median PFS was 12 months (95% CI, 11–13 months) in the STRT group, and 11 months (95% CI, 9–12 months) in the LTRT group (*P* = 0.136), and the OS was 36 months (95% CI, 4–67 months) versus 24 months (95% CI, 15–33 months), respectively (*P* = 0.071) (Fig. 2c and d). As shown in Table 3, patients with non-SL were associated with longer PFS (HR = 0.544, 95% CI, 0.343–0.863, *P* = 0.010) and OS (HR = 0.463, 95% CI, 0.256–0.837, *P* = 0.011) after controlling for confounding variables in multivariate analyses.

**Table 3:** Univariate and multivariate associations between patient characteristics and survival

Characteristic	Progression-free survival		Overall survival	
	HR (95% CI)	*P* value	HR (95% CI)	*P* value
Univariate associations				
Sex (female vs male)	0.752 (0.274–2.062)	0.579	0.357 (0.049–2.593)	0.308
Age (< 65 years vs ≥ 65 years)	0.690 (0.430–1.108)	0.125	0.880 (0.492–1.572)	0.880
ECOG performance status (0–1 vs 2)	1.930 (1.189–3.132)	0.008	1.152 (0.608–2.184)	0.665
Smoker (No vs others)	1.164 (0.662–2.045)	0.598	0.995 (0.496–1.997)	0.989
Histology (Squamous carcinoma vs others)	1.035 (0.653–1.642)	0.882	0.673 (0.366–1.238)	0.202
Stage				
IIIA vs IIIB	1.273 (0.655–2.476)	0.477	1.520 (0.624–3.705)	0.357
IIIA vs IIIC	0.914 (0.496–1.683)	0.773	1.056 (0.458–2.438)	0.898
Chemotherapy (concurrent vs sequent)	1.529 (0.905–2.584)	0.113	2.350 (1.185–4.663)	0.014
GTV (cm^3^)	1.002 (1.000–1.004)	0.014	1.004 (1.002–1.006)	< 0.001
PTV (cm^3^)	1.002 (1.001–1.003)	0.002	1.002 (1.001–1.004)	< 0.001
BED (Gy)	1.033 (0.969–1.101)	0.323	1.076 (0.994–1.164)	0.069
Baseline TLCs (cells/μL)	1.000 (0.999–1.000)	0.437	1.000 (0.999–1.001)	0.699
Pre-RT TLCs (cells/μL)	1.000 (0.999–1.000)	0.197	1.000 (0.999–1.000)	0.603
SL (Yes vs No)	0.524 (0.331–0.831)	0.006	0.460 (0.255–0.831)	0.010
TLCs nadir (cells/μL)	0.999 (0.998–1.000)	0.012	0.999 (0.997–1.000)	0.013
OTT (LTRT vs STRT)	0.671 (0.386–1.167)	0.158	0.519 (0.249–1.081)	0.080
IMRT (vs 3DCRT)	1.489 (0.881–2.517)	0.137	1.629 (0.850–3.122)	0.141
HT (vs 3DCRT)	1.072 (0.621–1.849)	0.803	0.987 (0.474–2.053)	0.972
Multivariate associations				
ECOG performance status (0–1 vs 2)	1.720 (1.052–2.811)	0.031	N/A	N/A
PTV (cm^3^)	1.002 (1000–1.003)	0.006	1.002 (1.001–1.004)	< 0.001
SL (Yes vs No)	0.544 (0.343–0.863)	0.010	0.463 (0.256–0.837)	0.011

*Abbreviations*: *HR* Hazard ratio, *CI* confidence interval, *ECOG* Eastern Cooperative Oncology Group, *GTV* gross tumor volume, *PTV* planning target volume, *BED* biological effective dose, *TLCs* total peripheral lymphocyte counts, *RT* radiotherapy, *SL* severe lymphopenia, *OTT* overall treatment time, Heart *V5* the percentage of total heart volume receiving at least 5 Gy, *LTRT* long-term radiotherapy, *STRT* short-term radiotherapy, *3DCRT* 3D conformal radiotherapy, *IMRT* Intensity modulated radiotherapy, *HT* Helical Tomotherapy, *N/A* not available

Note: Multivariate analysis includes sex, age, ECOG, and variables with a *P* values of ≤0.2 on univariate analysis

### Risk factors of SL

We further analyzed the association between TLC nadir and the dosimetric parameters of the lungs and heart (Additional file 2: Table S2). Because the parameters were all highly correlated with each other, we used ROC analyses to establish the best cut-off values and found that the mean lung dose (MLD) and heart V5 (the percentage of total heart volume receiving at least 5 Gy) were the best predictors of SL (area under the curve = 0.630 and 0.614, respectively) (Additional file 4: Figure S1). To avoid multicollinearity, MLD and heart V5 was included in the logistic regression analyses.

Considering the potential impact of other confounding factors on TLCs, including the aforementioned dosimetric parameters, we performed univariate and multivariate logistic analyses in the pooled cohort (Table 4). Specifically, univariate analyses showed that the development of SL was predictable by pre-RT TLCs (*P* <  0.001), GTV (*P* = 0.017), MLD (*P* = 0.013), Heart V5 (*s* = 0.016), and OTT (*P* = 0.007). On multivariate analysis, after controlling for confounding factors, greater GTV (OR = 6.909, 95% CI, 1.729–27.612; *P* = 0.006) and higher MLD (OR = 3.633, 95% CI, 1.349–9.787; *P* = 0.011) were significantly associated with an increased risk of developing SL, whereas higher pre-RT TLCs (OR = 0.998, 95% CI, 0.997–0.999; *P* <  0.001) and STRT (OR = 0.322, 95% CI, 0.115–0.908; *P* = 0.032) treatment were associated with a decreased risk of SL.

**Table 4:** Univariate and multivariate logistic regression of factors associated with SL during radiation treatment

Characteristic	OR (95% CI)	P
Univariate analysis		
Sex (female vs male)	0.814 (0.193–3.425)	0.778
Age (< 65 years vs ≥ 65 years)	1.124 (0.531–2.382)	0.760
ECOG (0–1 vs 2)	1.439 (0.621–3.334)	0.395
Underlying respiratory system disease (No versus Yes)	1.586 (0.744–3.383)	0.233
Smoker (No vs others)	1.275 (0.518–3.139)	0.597
Induction chemotherapy (No vs Yes)	2.432 (0.963–6.142)	0.06
Concurrent chemotherapy (No vs Yes)	0.703 (0.308–1.607)	0.404
Systemic Corticosteroids (No vs Yes)^a^	0.867 (0.357–2.106)	0.752
Pre-RT TLCs (cells/μL)	0.998 (0.998–0.999)	< 0.001
GTV (< 22 cm^3^vs ≥ 22 cm^3^)	3.867 (1.276–11.714)	0.017
PTV(< 48 cm^3^ vs ≥ 48 cm^3^)	1.478 (0.464–4.706)	0.509
Mean lung dose (< 1150 cGy vs ≥ 1150 cGy)	2.720 (1.239–5.969)	0.013
Heart V5 (< 6 vs ≥ 6)	3.704 (1.273–10.777)	0.016
BED (Gy)	1.026 (0.927–1.137)	0.616
OTT (LTRT vs STRT)	0.289 (0.117–0.715)	0.007
IMRT (vs 3DCRT)	1.048 (0.412–2.665)	0.922
HT (vs 3DCRT)	0.829 (0.353–1.946)	0.667
Stage IIIB (vs IIIA)	0.900 (0.299–2.712)	0.851
Stage IIIC (vs IIIA)	0.884 (0.325–2.403)	0.809
Multivariate analysis^b^		
Pre-RT TLCs (cells/μL)	0.998 (0.997–0.999)	< 0.001
GTV (< 22 cm^3^vs ≥ 22 cm^3^)	6.909 (1.729–27.612)	0.006
Mean lung dose (< 1150 cGy vs ≥ 1150 cGy)	3.633 (1.349–9.787)	0.011
Treatment duration (LCRT vs SCRT)	0.322 (0.115–0.908)	0.032

*Abbreviations*: *SL* severe lymphopenia, *OR* Odds ratio, *CI* confidence interval, *ECOG* Eastern Cooperative Oncology Group, *RT* radiotherapy, *TLCs* total lymphocyte counts, *GTV* gross tumor volume, *PTV* planning target volume, *Heart V5* the percentage of total heart volume receiving at least 5 Gy, *BED* biological effective dose, *OTT* overall treatment time, *LTRT* long-term radiotherapy, *STRT* short-term radiotherapy, *3DCRT* 3D conformal radiotherapy, *IMRT* Intensity modulated radiotherapy, *HT* Helical Tomotherapy

Note: ^a^equivalent to approximately 60–120 mg of prednisone

^b^Multivariate analysis includes sex, age, ECOG, induction chemotherapy, Pre-RT TLCs, GTV, mean lung dose, Heart V5, and treatment duration

Since the RT technique could confound the choice of fractionation, a subgroup multivariate analysis was also conducted for patients treated with helical tomotherapy (HT). Similar to the whole cohort, STRT (OR = 0.223, 95% CI, 0.056–0.887; *P* = 0.033) was significantly associated with a decreased risk of developing SL after controlling for confounding factors (Additional file 3: Table S3).

## Discussion

Many studies have shown that RT can dramatically reduce TLCs, and that low TLC nadirs were correlated with poor survival in many solid tumors. Our results further suggested that the level of TLCs was related to the RT treatment duration for unresectable stage III NSCLC. TLCs declined steeply each week for the first 5 weeks, after which, TLCs nadir occurred at approximately the 5th week. Increasing the dose of radiation per fraction to deliver the entire RT regimen in 4 weeks could significantly lower the risk of developing SL. This is in line with a recent study suggesting that SBRT could decrease the severity of RIL compared to CFRT in locally advanced pancreatic cancer [18]. Finally, our results revealed a significantly reduced risk of disease progression and death in patients who did not experience SL during RT.

Radiation can suppress or stimulate the immune system. The contribution of lymphocytes to radiation-induced tumor control was shown in mouse models over 30 years ago [19], and more recently, the availability of T cell receptor (TCR)-transgenic mice made it possible to unequivocally demonstrate that radiation can induce priming of T cells to exogenous model antigens expressed by tumors [20, 21]. Those studies together with the demonstration that radiation induces immunogenic cell death [22], have provided proof that radiation can induce tumor-specific T cells. This study revealed an association between higher lymphocyte levels during treatment and better clinical outcomes. Maintaining an intact adaptive immune system during cancer therapy may be important for enhancing the effectiveness of cytotoxic therapies and improving cancer control. This may impact cancer recurrence by affecting the numbers of tumor-infiltrating lymphocytes, which correlates with the prognosis of multiple cancers [23, 24]. This is consistent with our observation that TLC nadir was associated with worse PFS and OS.

The clinical implications of our findings may also be more pronounced in the setting of the new therapeutic strategy of combining RT and immunotherapy in NSCLC patients. As promising results were observed in the PACIFIC trial, consolidation treatments with immune checkpoint inhibitors (ICIs) are now recommended for patients with stage III, unresectable NSCLC after receiving definitive CRT [16]. Previous work has shown that RIL would further compromise the therapeutic efficacies of ICIs through the loss of effector cells, which identify and destroy tumor cells [25, 26]. The efficacy of ICIs relies on the modulation of lymphocyte activity and are dependent on circulating lymphocyte counts [27]. SL at the onset of ICI was independently associated with poor survival [25]. This further emphasized the importance of preserving and maintaining circulating lymphocytes in the emerging era of clinical immunotherapies. Considering the potential therapeutic abilities of circulating lymphocyte populations, a hypofractionated regimen with fewer fractionations and shorter treatment course (within 4 weeks) may be a superior approach for combination with immunotherapies.

Pulmonary circulation is the main portion of the cardiovascular system in which oxygen-depleted blood is pumped away from the heart via the pulmonary artery. A separate system known as bronchial circulation also supplies blood to the tissues of the larger airways. Arteries are further divided into very fine capillaries, which are extremely thin-walled and cover the lung. Some tumors may also be adjacent to heart, thoracic aorta, and vein which are filled with blood. Thus, the distribution of low-dose irradiation could include part of the heart or large vessels. One proposed mechanism for RIL is via radiation exposure of the circulating blood pool, since lymphopenia occurs after irradiation of tissues, including breast and brain, which contain little bone marrow or lymphatic tissues, respectively [28, 29]. Peripheral blood lymphocytes are known to be extremely sensitive to radiation despite mitotic inactivity [30]. Nevertheless, an unexpected finding of our study was that the TLC nadirs occurred at approximately the fifth week, not at the completion of RT or upon delivery of maximal doses.

Multiple studies demonstrated that pretreatment with low doses of irradiation can induce resistance to damage from subsequent high doses of irradiation [31, 32]. Yovino et al. analyzed a modeled “typical” radiation plan for glioblastoma (60 Gy/30 fractions) and found that a single fraction caused 0.5 Gy exposure to 5% of all circulating blood cells. L. Basler et al. also regarded that the proportion of circulating lymphocytes exposed to at least 0.5 Gy was a surrogate parameter for radiation-induced immunosuppression [33]. The circulating blood dose appeared to depend on the target volume and fraction number in early treatments. However, as treatment progressed to the 5th week of RT, nearly all the circulating blood cells were projected to receive at least 0.5 Gy (mean dose 2.2 Gy) [16]. This simulation model might explain our finding that by the 5th week of RT, nearly all circulating lymphocytes had received a low-dose (at least 0.5 Gy) of irradiation and developed resistance. The underlying mechanisms of this phenomenon are not well understood and our findings provide an important basis for further investigations.

In addition to the treatment duration, pre-RT TLCs, GTV, and MLD were also found to strong impact RIL in our study. A higher GTV and MLD tended to trigger greater depletion of peripheral lymphocytes due to greater exposure of those lymphocytes to radiation. We had excluded patients who had received any other anticancer treatments besides CRT before disease progression, had used any systemic corticosteroids during RT except for pretreatment before chemotherapy, had any ongoing infection, rheumatoid disease, or immune system disease to eliminate potential clinical factors influencing the lymphopenia. As for underlying respiratory system disease, our data show that it has no impact on lymphopenia. The contribution of chemotherapy to lymphopenia has been disputed [14, 34]. Our study showed that concurrent chemotherapy was not associated with SL, which was consistent with previous study [14]. Additionally, there was no difference in the median baseline TLCs or pre-RT TLCs in our study population, whether stratified by the chemotherapy regimen or cycles (all *P* > 0.05). This was consistent with the notion that the choice of chemotherapy regimens and the cycles had no correlation with lymphopenia in patients with stage III NSCLC [35]. Thus, the chemotherapy regimen used in our study population did not play a major role in causing lymphopenia. Bone marrow suppression caused by chemotherapy primarily resulted in the reduction of peripheral neutrophil cell counts in our patients (data not shown). Given the potential effect of corticosteroids on circulating lymphocytes, patients who had received systemic steroids during RT were excluded from our analyses, unless steroids were used as pretreatments before chemotherapy [36, 37]. However, logistic regression analyses showed that the dose of corticosteroids (equivalent to approximately 60–120 mg of prednisone) was not associated with an increased risk of developing SL. Clinical trials for ICIs typically excluded patients who received systemic steroids exceeding 10 mg of prednisone daily (or the equivalent) [38]. Collectively, our findings indicated that the doses of transient corticosteroid administration and chemotherapy had no obvious cytotoxicity to circulating lymphocytes.

Several limitations should be noted in interpreting our results. First, the frequencies of blood tests during RT were subject to variations, although the general practice at our institution was to perform blood testing prior to, and weekly during, RT. Second, considering the TLC nadir was at the 5th week of RT, we divided patients into two groups based on whether the RT course was completed within 4 weeks. However, the small number of patients in the STRT group decreased the analysis power. For example, despite a large absolute difference in 3-year OS rates favoring the STRT group after adjustment, the data did not reach statistical significance. Third, the selection of fractionation and dosing schemes was generally based on the treating physician’s preference. Fourth, unscheduled treatment interruptions due to malfunctions prolonged the OTT. Therefore, we cannot exclude the possibility that such occurrences biased the results.

## Conclusions

In summary, our observations suggest that changes in TLCs during definitive RT was related to treatment-durations in unresectable stage III NSCLC. TLC nadirs occurred at approximately the fifth week of RT, not upon completion of RT or upon administration of the maximal dose. Decreasing the total number of RT fractions and increasing the dose of each fraction to complete treatment within 4 weeks while maintaining same overall dose is a potential strategy to protect circulating lymphocytes and improve clinical outcomes. The clinical implications of our findings may be more pronounced in the setting of a new therapeutic strategy combining RT and immunotherapy for unresectable stage III NSCLC, particularly given the promising results observed in the PACIFIC trial. Additional large-scale prospective investigations are needed to test the potential lymphocyte-sparing of hypofractionation, particularly when used in combination with immunotherapeutic approaches.

## Additional files

Additional file 1: **Table S1.** Details of chemotherapy used in our study population (*n* = 115). (DOCX 16 kb)

Additional file 2: **Table S2.** Correlation between TLCs nadir and percentage of lung or heart dose. (DOCX 14 kb)

Additional file 3: **Table S3.** Multivariate logistic regression associating baseline variables with SL during radiation treatment in subgroup patients treated with helical tomotherapy. (DOCX 14 kb)

Additional file 4: **Figure S1.** Receiver operating characteristic (ROC) curves of mean lung dose and heart V5 (the percentage of total heart volume receiving at least 5 Gy). (DOCX 16 kb)

## Abbreviations

BED: Biological effective dose
CFRT: Conventionally-fractionated radiotherapy
CRT: Chemoradiation therapy
ECOG: Eastern Cooperative Oncology Group
EQD2: Equivalent dose in 2 Gy fraction
GTV: Gross target volume
HFRT: Hypofractionated radiotherapy
HR: Hazard ratio
HT: Helical tomotherapy
ICIs: Immune checkpoint inhibitors
IQR: Interquartile range
LTRT: Long-term radiotherapy
MHD: Mean heart dose
MLD: Mean lung dose
NSCLC: Non-small cell lung cancer
OR: Odds ratio
OS: Overall survival
OTT: Overall treatment times
PET/CT: Positron emission tomography/computed tomography
PFS: Progression-free survival
PTV: Planning target volume
RIL: Radiation-induced lymphopenia
ROC: Receiver operating characteristic
RT: Radiation therapy
SL: Severe lymphopenia
STRT: Short-term radiotherapy
TCR: T cell receptor
TLCs: Total peripheral lymphocyte counts
