# Supplementary material for: Treatment-duration is related to changes in peripheral lymphocyte counts during definitive radiotherapy for unresectable stage III NSCLC
Source: Radiat Oncol. 2019 May 27;14:86. doi: 10.1186/s13014-019-1287-z (PMC6537222; doi:10.1186/s13014-019-1287-z)
Supplement: Supplementary file 1 — Table S1. Details of chemotherapy used in our study population (n = 115). (DOCX 16 kb) [file 13014_2019_1287_MOESM1_ESM.docx]

Table S1. Details of chemotherapy used in our study population (n = 115).

| Induction chemotherapy | No. of cases (%) |
| --- | --- |
| Paclitaxel (Albumin Bound) | 1 (0.9%) |
| Paclitaxel | 32 (27.8%) |
| Docetaxel | 19 (16.5%) |
| Pemetrexed | 18 (15.7%) |
| Gemcitabine | 18 (15.7%) |
| Vinorelbine | 2 (1.7%) |
| Etoposide | 1 (0.9%) |
| Concurrent chemoradiotherapy |  |
| Paclitaxel (Albumin Bound) | 2 (1.7%) |
| Paclitaxel | 12 (10.4%) |
| Docetaxel | 3 (2.6%) |
| Pemetrexed | 10 (8.7%) |
| Etoposide Phosphate | 1 (0.9%) |
| Platinum weekly | 3 (2.6%) |
| Sequential chemoradiotherapy |  |
| Pemetrexed | 14 (12.2%) |
| Paclitaxel | 33 (28.7%) |
| Docetaxel | 21 (18.3%) |
| Gemcitabine | 2 (1.7%) |
| Vinorelbine | 2 (1.7%) |
| Unknown | 12 (10.4%) |
