# Supplementary material for: Treatment-duration is related to changes in peripheral lymphocyte counts during definitive radiotherapy for unresectable stage III NSCLC
Source: Radiat Oncol. 2019 May 27;14:86. doi: 10.1186/s13014-019-1287-z (PMC6537222; doi:10.1186/s13014-019-1287-z)
Supplement: Supplementary file 2 — Table S2. Correlation between TLCs nadir and percentage of lung or heart dose. (DOCX 14 kb) [file 13014_2019_1287_MOESM2_ESM.docx]

Table S2. Correlation between TLCs nadir and percentage of lung or heart dose.

| Dosimetric parameters | spearman correlation coefficients (r) | P value |
| --- | --- | --- |
| Mean lung dose | -0.191 | 0.041 |
| Lung V5 | -0.236 | 0.055 |
| Lung V10 | -0.222 | 0.071 |
| Lung V15 | -0.230 | 0.061 |
| Lung V20 | -0.161 | 0.194 |
| Lung V25 | -0.126 | 0.311 |
| Lung V30 | -0.095 | 0.477 |
| Lung V35 | -0.070 | 0.575 |
| Lung V40 | -0.065 | 0.603 |
| Lung V45 | -0.061 | 0.624 |
| Lung V50 | -0.019 | 0.878 |
| Lung V55 | -0.005 | 0.969 |
| Lung V60 | -0.085 | 0.492 |
| Mean heart dose | -0.227 | 0.041 |
| Heart V5 | -0.182 | 0.156 |
| Heart V10 | -0.239 | 0.061 |
| Heart V15 | -0.255 | 0.046 |
| Heart V20 | -0.248 | 0.052 |
| Heart V25 | -0.259 | 0.042 |
| Heart V30 | -0.254 | 0.047 |
| Heart V35 | -0.246 | 0.054 |
| Heart V40 | -0.226 | 0.078 |
| Heart V45 | -0.243 | 0.057 |
| Heart V50 | -0.229 | 0.073 |
| Heart V55 | -0.182 | 0.157 |
| Heart V60 | -0.023 | 0.857 |

Vn (%), the percentage of total lung or heart volume receiving at least n dose of radiation.
