# Supplementary material for: Treatment-duration is related to changes in peripheral lymphocyte counts during definitive radiotherapy for unresectable stage III NSCLC
Source: Radiat Oncol. 2019 May 27;14:86. doi: 10.1186/s13014-019-1287-z (PMC6537222; doi:10.1186/s13014-019-1287-z)
Supplement: Supplementary file 3 — Table S3. Multivariate logistic regression associating baseline variables with SL during radiation treatment in subgroup patients treated with helical tomotherapy. (DOCX 14 kb) [file 13014_2019_1287_MOESM3_ESM.docx]

Table S3. Multivariate logistic regression associating baseline variables with SL during radiation treatment in subgroup patients treated with helical tomotherapy

| Characteristic | OR (95% CI) | P |
| --- | --- | --- |
| Pre-RT TLCs | 0.998 (0.997-1.000) | 0.036 |
| Induction chemotherapy | 7.312 (1.461-36.591) | 0.015 |
| OTT (STRT vs LTRT) | 0.223 (0.056-0.887) | 0.033 |

Abbreviations: SL, severe lymphopenia; OR, Odds ratio; CI, confidence interval; RT, radiotherapy; TLCs, total lymphocyte counts; OTT, overall treatment time; LTRT, long-term radiotherapy; STRT, short-term radiotherapy.

Note: Multivariate analysis includes sex, age, ECOG, induction chemotherapy, Pre-RT TLCs, GTV, mean lung dose, Heart V5, and treatment duration.
